# Findings from a Genotyping Study of over 1000 People with Inherited Retinal Disorders in Ireland

**DOI:** 10.3390/genes11010105

**Published:** 2020-01-16

**Authors:** Laura Whelan, Adrian Dockery, Niamh Wynne, Julia Zhu, Kirk Stephenson, Giuliana Silvestri, Jacqueline Turner, James J. O’Byrne, Matthew Carrigan, Peter Humphries, David Keegan, Paul F. Kenna, G. Jane Farrar

**Affiliations:** 1 The School of Genetics & Microbiology, Trinity College Dublin, D02 VF25 Dublin, Ireland; dockerya@tcd.ie (A.D.); carrigma@tcd.ie (M.C.); pete.humphries@tcd.ie (P.H.); Paul.Kenna@tcd.ie (P.F.K.); gjfarrar@tcd.ie (G.J.F.); 2 The Research Foundation, Royal Victoria Eye and Ear Hospital, D02 XK51 Dublin, Ireland; wynnenc@tcd.ie; 3 Clinical Genetics Centre for Ophthalmology, The Mater Misericordiae University Hospital, D07 R2WY Dublin, Ireland; julia.q.zhu@gmail.com (J.Z.); kirkstephenson@hotmail.com (K.S.); jturner@mater.ie (J.T.); jamesobyrne@mater.ie (J.J.O.); dkeegan@mater.ie (D.K.); 4 Department of Ophthalmology, The Royal Victoria Hospital, Belfast BT12 6BA, Northern Ireland, UK; Julie.Silvestri@belfasttrust.hscni.net; 5 Centre for Experimental Medicine, Queen’s University Belfast, Belfast BT7 1NN, Northern Ireland, UK

**Keywords:** inherited eye disease, ophthalmic genetics, genomics, next generation sequencing, retinopathy, rare variants, novel variants

## Abstract

The Irish national registry for inherited retinal degenerations (Target 5000) is a clinical and scientific program to identify individuals in Ireland with inherited retinal disorders and to attempt to ascertain the genetic cause underlying the disease pathology. Potential participants first undergo a clinical assessment, which includes clinical history and analysis with multimodal retinal imaging, electrophysiology, and visual field testing. If suitable for recruitment, a sample is taken and used for genetic analysis. Genetic analysis is conducted by use of a retinal gene panel target capture sequencing approach. With over 1000 participants from 710 pedigrees now screened, there is a positive candidate variant detection rate of approximately 70% (495/710). Where an autosomal recessive inheritance pattern is observed, an additional 9% (64/710) of probands have tested positive for a single candidate variant. Many novel variants have also been detected as part of this endeavor. The target capture approach is an economic and effective means of screening patients with inherited retinal disorders. Despite the advances in sequencing technology and the ever-decreasing associated processing costs, target capture remains an attractive option as the data produced is easily processed, analyzed, and stored compared to more comprehensive methods. However, with decreasing costs of whole genome and whole exome sequencing, the focus will likely move towards these methods for more comprehensive data generation.

## 1. Introduction

Inherited retinal degenerations (IRDs) are a broad set of clinically and genetically diverse conditions that represent the leading cause of visual dysfunction in those of working age. IRDs are typically caused by improper development or death of photoreceptor cells and have a substantial effect on both the quality of life of those affected and health economics. Inheritance patterns include autosomal recessive, autosomal dominant and X-linked, as well as rarer mitochondrial and digenic forms [1,2]. Since the discovery of the first IRD genes via linkage analysis [3,4,5], a comprehensive repertoire of disease-causing genes has been described.

To date, over 270 genes have been associated with a wide variety of IRDs across a phenotypic spectrum [6], which are routinely divided into large subcategories in order to readily distinguish the specific retinal regions and cell types affected; for example, retinitis pigmentosa (RP) versus macular dystrophy, which initially cause predominantly peripheral and central vision loss, respectively. In the majority of IRDs, ocular manifestations occur in isolation. However, many forms of syndromic IRD that result in systemic disease have also been described [7]; more common forms include Usher Syndrome and Bardet-Biedl syndrome. RP is the most frequent IRD presentation with an incidence of approximately 1 in 3000 [8]. Over 70 genes are implicated as causative of RP, making it one of the most diverse and genetically heterogeneous Mendelian conditions described to date [9]. Other IRDs are less heterogeneous—autosomal recessive Stargardt disease, for example, which is caused by pathogenic variations in *ABCA4* in approximately 95% of cases [10,11,12,13]. Furthermore, IRDs such as X-linked retinoschisis are characterised by a single gene aetiology [14].

Using the incidence rates previously mentioned, it can be estimated that over 2.5 million individuals are affected by RP alone worldwide, and diagnoses are often based solely on clinical presentation [15]. The importance of identifying the genetic causes of IRDs is becoming increasingly clear, allowing for more accurate prognosis of disease progression and risk assessment for other family members. Importantly, a genetic diagnosis can also facilitate a better understanding of the disease for those affected. Furthermore, multiple clinical trials to treat these diseases are ongoing, with the first gene therapy for IRD, Luxturna, having been approved for use by both the US Food and Drug Administration and the European Medicines Agency [16]. Typically, such trials and treatments require that a causative variant has been identified in order to participate. Here, we present an update on Target 5000, a next-generation sequencing (NGS) study that aims to provide a large cohort of Irish patients with an accurate genetic diagnosis, whilst further elucidating the underlying genetic architecture of IRDs in Ireland.

Target 5000 is the Irish national registry for those affected by an IRD. It is both a research and clinical study that aims to elucidate the underlying genetic architecture of IRDs in Ireland and to provide an accurate genetic diagnosis for those affected. Target 5000 utilises target capture next generation sequencing, a time and cost-effective screening method that boasts more even coverage across captured regions, positively affecting diagnostic yield, in comparison with whole exome sequencing (WES) [17]. Exonic regions of over 250 IRD-associated genes as well as a number of intronic regions harbouring previously identified pathogenic variants were captured. Although whole genome sequencing (WGS) has become more accessible in recent years and facilitates the discovery of both deep intronic variants and novel genotype-phenotype correlations [18], significant challenges remain in terms of data handling, variant interpretation, and cost. Target 5000 significantly highlights the benefits of target capture NGS as a preliminary diagnostic measure, before employing more comprehensive sequencing methods such as WES or WGS.

Here we present novel findings from both newly recruited participants and retrospective analysis of 750 individuals previously screened, resulting over 1000 individuals across 710 pedigrees genetically screened to date. 458 individuals have been sequenced for variants in 210 genes, 689 individuals have been sequenced for variants in 254 genes, and confirmation sequencing is ongoing for other affected family members. The average age of recruited participants to Target 5000 is 55 (Figure S1). Target 5000 has identified positive candidate variants in almost 70% (495/710) of cases, including both previously reported pathogenic and novel potentially pathogenic variants. This includes novel structural variants, single nucleotide variants, and splice variants. Furthermore, 9% (64/710) of pedigrees were found to carry a pathogenic/likely pathogenic variant on one allele where the genotype and phenotype were associated with a recessive inheritance pattern.

## 2. Materials and Methods

### 2.1. Patient Identification and Recruitment

Probands and other family members are recruited nationwide and primarily assessed at the Research Foundation of the Royal Victoria Eye and Ear Hospital (Dublin, Ireland), the Mater Misericordiae University Hospital (Dublin, Ireland), and the Royal Victoria Hospital (Belfast, Northern Ireland, UK). This allows for the inclusion of participants from both The Republic of Ireland and Northern Ireland. Following informed consent, the applicable combination of tests were carried out to accurately assess the patients’ phenotype (Table 1).

### 2.2. DNA Acquisition and Next Generation Sequencing

DNA was isolated from either blood (DNA Blood Maxi Kit, Qiagen, Hilden, Germany) or saliva (Oragene-DNA, DNA Genotek, ON, Canada) samples from participants. Sample preparation was carried out using a hybridisation-based target capture sequencing method previously described [15]. The average read coverage achieved was 125× per captured region. All genomic locations refer to the Hg38 reference genome (Homo sapiens GRCh38).

### 2.3. Variant Confirmation Sequencing

To validate variants identified by NGS, relevant genomic loci containing the mutations were amplified by polymerase chain reaction (PCR) and investigated by direct sequencing. Primers were procured from Sigma-Aldrich (Gillingham, England, UK). DNA products were standardly amplified using Q5 High-Fidelity 2× Master Mix (New England Biolabs Inc., Ipswich, MA, USA). Segregation analyses for additional family members were amplified similarly or alternatively, directly from blood (Phusion Blood Direct PCR Kit, Thermo Scientific, MA, USA) or saliva (Phusion Human Specimen Direct PCR Kit, Thermo Scientific) where applicable. The annealing temperatures for reactions were optimised for each variant; all other details were executed as per the supplier’s recommendations. Sanger sequencing was performed by Eurofins Genomics (Ebersberg, Germany).

### 2.4. Choroideremia Deletion Confirmation

Primers were designed to probe for the exonic regions of *CHM* (MIM: 300390; ChrX: 85861180-86047558) and the neighbouring genes, *POF1B* (MIM: 300603; ChrX: 85277396-85379709) and *DACH2* (MIM: 300608; ChrX: 86148451-86832602), as well as the intergenic space between. A full list of the primers utilised is available in Supplementary Materials (Table S1).

### 2.5. Sequencing of RPGR ORF15

A previously reported sequencing strategy of RPGR was employed in order to cover the highly repetitive sequences of ORF15, a major cause of X-linked RP [19]. Primers used to probe this region can be found in Figure S2.

### 2.6. Single-Molecule Molecular Inversion Probe (smMIP)-Based Sequencing of ABCA4

smMIPs-based whole gene sequencing of *ABCA4* was carried out using a NextSeq 500 as part of a large-scale study by Khan et al. [13]. 3866 single-molecule molecular inversion probes (smMIPs) were designed as described previously [20] and employed to capture 110nt increments of both the sense and antisense strands of the *ABCA4* gene, as well as 40 kb of flanking sequences. Sample processing, sequencing, data analysis, and variant interpretation was carried out, as described by Khan et al. [13].

### 2.7. Data Analysis and Variant Interpretation of Target Capture NGS Data

Raw sequencing data were demultiplexed and mapped to the IRD-relevant regions of the human genome (Hg38) as previously described [15]. The American College of Medical Genetics and Genomics (ACMG) criteria for classifying pathogenic variants was utilised to interpret variants [21]. Other proposed modifications to the ACMG guidelines to further quantitate certain lines of evidence were also employed during the study. These modifications effect three lines of evidence that can be applied. These adjustments consider using segregation data in a quantitative manner (code: PP1) [22], quantitating evidence that considers the rare incidence of variants (code: PS4) and the value of accurately phenotyping conditions with single gene aetiologies (code: PP4) [23]. To further implement these recommendations, REVEL and dbscSNV RF scores were added to the existing suite of ensemble predictors to more stringently detect agreement between predictors of pathogenicity [24,25]. Similarly, Manta was added to our structural variant pipeline to assist in the detection of genomic rearrangements [26].

### 2.8. Ethical Approval

Ethical approval for this study was awarded by the Research and Medical Ethics committee of the Royal Victoria Eye and Ear Hospital (13-06-2011: HRA-POR201097) and by the Institutional Review Board of the Mater Misericordiae University Hospital and Mater Private Hospital (MMUH IRB 1/378/1358), Dublin, Ireland, prior to commencement. All work was carried out in accordance with the approved guidelines. All patients have given informed consent before recruitment to the study.

## 3. Results

### 3.1. Clinical Presentation and Positive Candidate Detection Rates

**Figure 1:**
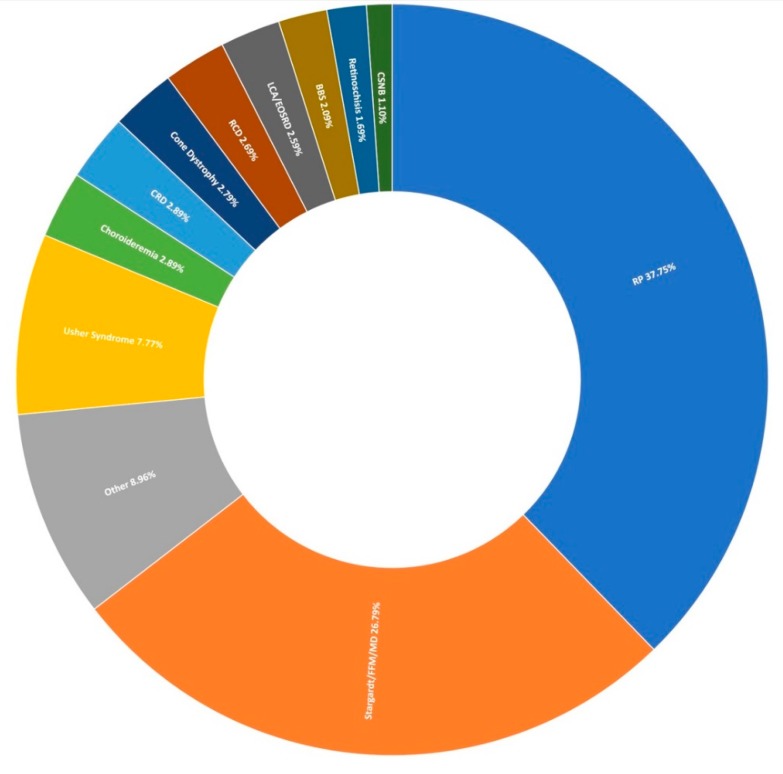
Clinical presentation of 1004 recruited Target 5000 pedigrees. RP: Retinitis Pigmentosa, FFM: Fundus Flavimaculatus, MD: Macular Dystrophy, CRD: Cone-Rod Dystrophy, RCD: Rod-Cone Dystrophy, LCA: Leber Congenital Amaurosis, EOSRD: Early-Onset Retinal Degeneration, BBS: Bardet-Biedl Syndrome, CSNB: Congenital Stationary Night Blindness (Table S2).

**Figure 2:**
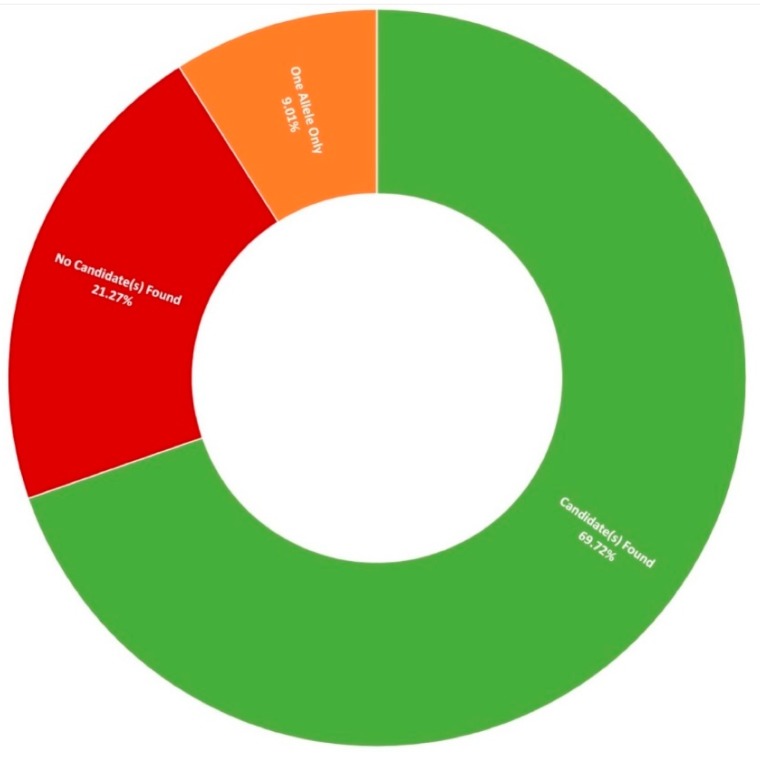
Diagnostic yield rates for 710 Target 5000 pedigrees utilising target capture next-generation sequencing of the exonic regions of over 250 genes and previously identified pathogenic intronic variants (Table S3).

Thus far, 710 pedigrees have been analysed as part of this study by target capture sequencing. 458 individuals have been sequenced for variants in 210 genes, 689 individuals have been sequenced for variants in 254 genes, and confirmation sequencing is ongoing for other affected family members. A spectrum of IRDs were observed in this study, as can be seen in Figure 1. The positive candidate dectection rate has improved from previous reports [15,27], now reaching approximately 70% (495/710). Furthermore, a single candidate variant only has been identified in an additional 9% (64/710) of pedigrees diagnosed with an IRD that typically exhibits a recessive inheritance pattern (Figure 2). In addition, a number of novel missense variants have been detected in the course of this study (Table 2).

### 3.2. Retinitis Pigmentosa

**Figure 3:**
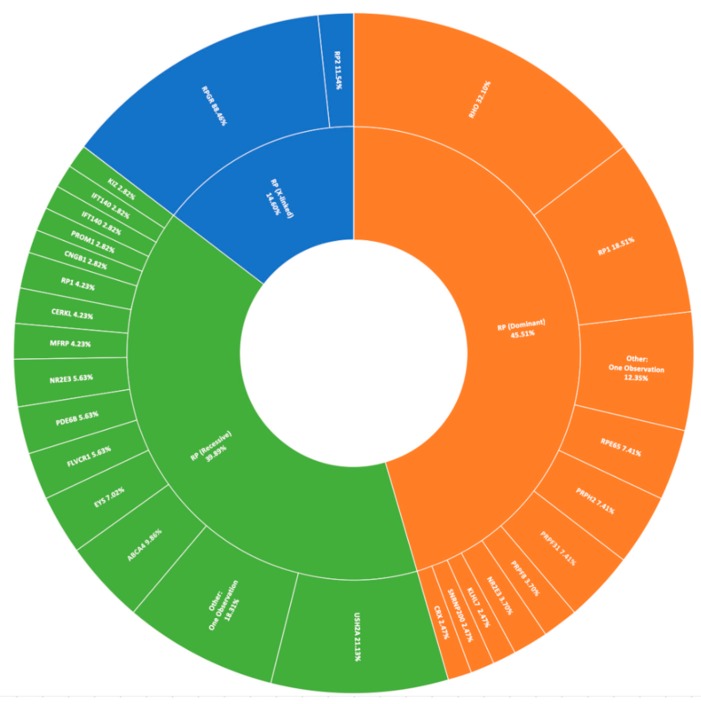
A summary of the genetic architecture of retinitis pigmentosa (RP) in Target 5000 participants (Table S4).

The most prevalent clinical presentation was retinitis pigmentosa (RP; MIM: 268000), accounting for 37.75% (379/1004) of all recruited pedigrees (Figure 1). These figures incorporate various phenotypic presentations, including atypical, inverse, and paravenous RP. The total number of pedigrees analysed and clinically reported as having a family history of RP was 184. Of these, 131 have been genetically resolved. The total number of pedigrees analysed and clinically reported as having simplex RP was 79. 48 of these pedigrees have been genetically resolved. This gives a genetic diagnosis rate of 71% and 61%, respectively. Of these resolved cases, 81 exhibited an autosomal dominant inheritance pattern, 71 exhibited an autosomal recessive inheritance pattern, and 27 exhibited an X-linked inheritance pattern. Autosomal dominant was the most common inheritance pattern, accounting for over 45% (81/178) of all sequenced RP pedigrees in this cohort (Figure 3). This figure is larger than reported in other studies [28]. Variants in *RHO* (MIM: 180380) were the most common candidate variants for RP in this cohort, accounting for over 14% (26/178) of all sequenced RP pedigrees, and over 32% (26/81) of dominant pedigrees alone. It is important to note that there is likely an even higher prevalence of *RHO*-linked RP in the Irish IRD cohort, as many pedigrees involved in single-gene studies were excluded from this study due to the discovery of a causative variant previously [15,27]. Of these variants, namely c.533A>G (p.Tyr178Cys), c.541G>A (p.Glu181Lys), and c.620T>G (p.Met207Arg) collectively account for candidate *RHO* mutations across 13 different pedigrees to date.

**Figure 4:**
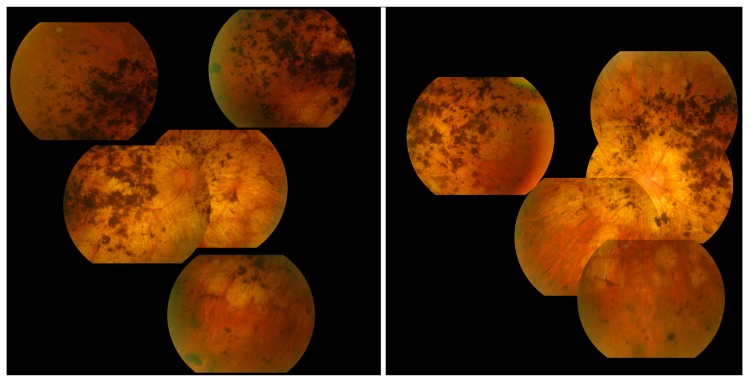
Collage of fundus images of a patient initially presenting with a Stargardt phenotype that progressed to phenotype suggestive of retinitis pigmentosa. Extensive pigmentation can be seen at both maculae and also in the periphery of both eyes (**left**—right eye, **right**—left eye). There are also distinct punched-out areas in the pigment epithelium bilaterally.

**Figure 5:**
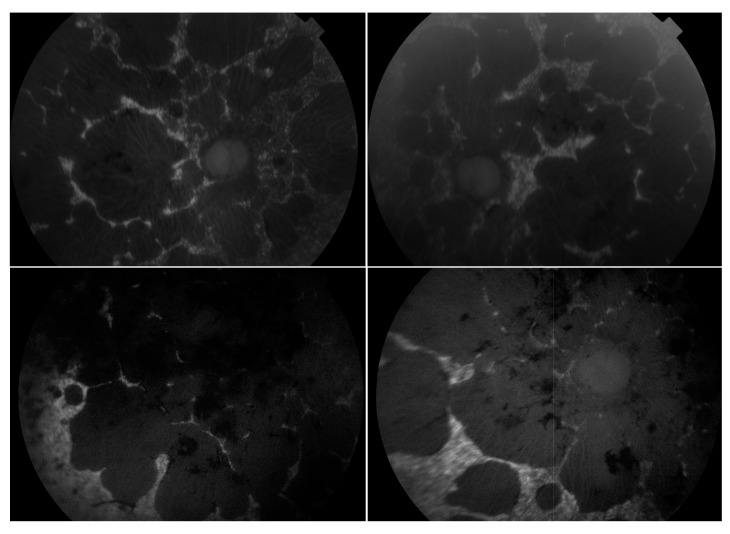
Montage of autofluorescence fundus images of the patients initially presenting with a Stargardt phenotype that progressed to phenotype suggestive of retinitis pigmentosa. The right eyes are shown in the **left** column; the left eyes are shown in the **right** column. Patient example 1 is the first row and patient example 2 is the second row. All eyes demonstrate a dark choroid and large nummular regions of hypoautofluorescence throughout the retinae.

The most commonly observed candidate gene for autosomal recessive RP was *USH2A* (MIM: 608400). Two variants in this gene were found in over 21% (15/71) of pedigrees with this phenotype (Figure 3). As variants in *USH2A* are also the primary cause of Type II Usher Syndrome, it is important to note that participants included in this section presented with recessive RP without syndromic disease manifestation. Non-syndromic *USH2A*-linked RP has been consistently reported in other IRD cohorts [29]. Interestingly, a proportion of recessive RP pedigrees (>9%) (7/71) carry variants in *ABCA4*, typically associated with Stargardt Disease, a form of macular dystrophy. In the Target 5000 patient cohort, it has become apparent that an atypical RP phenotype appears to be caused by variants in *ABCA4*, as previously described in other cohorts (Figure 4 and Figure 5) [10,30].

Pathogenic variants in *RPGR* (MIM: 312610) were the most frequent cause of X-Linked RP, explaining over 88% (23/26) of sequenced pedigrees (Figure 3). Employment of a bespoke sequencing strategy of ORF15 has enhanced the success of sequencing this region, which typically presents a diagnostic challenge due to low coverage and poor variant detection [19].

### 3.3. Stargardt Disease and Other Macular Dystrophies

**Figure 6:**
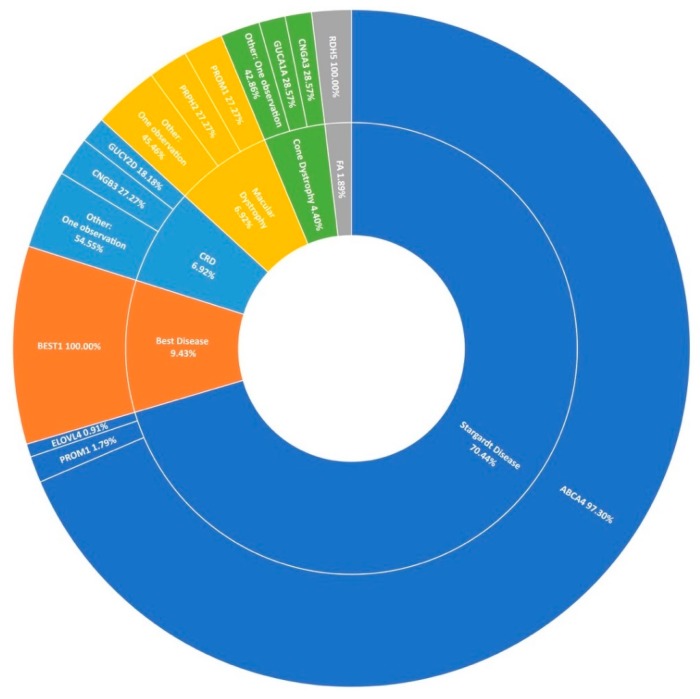
A summary of the genetic architecture of macular dystrophies in Target 5000 participants. CRD: Cone-Rod Dystrophy FA: Fundus Albipunctatus (Table S5).

Stargardt disease (STGD1) is the second largest phenotypic presentation in this study (Figure 1). As mentioned previously, variants in *ABCA4* [31] were the most common cause, accounting for over 97% (109/112) of resolved STGD1 pedigrees in this cohort (Figure 6). Six potentially novel coding variants in *ABCA4* were detected as part of this study, NM_000350.2: c.1865G>A (p.Ser622Asn), c.223T>C (p.Cys75Arg), c.4468T>C (p.Cys1490Arg), c.5329A>G (p.Met1777Val), c.5351T>C (p.Leu1784Pro), and c.6743T>C (Phe2248Ser). All of these variants, with the exception of c.5351T>C (p.Leu1784Pro), were observed in individual pedigrees where another known pathogenic *ABCA4* variant was also observed. Variant c.5351T>C (p.Leu1784Pro) was observed in three individuals of two not knowingly related families also carrying a known pathogenic variant. The probands of these pedigrees do not appear to share any other rare variants in common in the regions captured.

It is becoming increasingly clear that intronic variants play a major role in the causation of STGD1. Nine pathogenic intronic variants were included in the capture panel of this study and found in 26 cases in 20 pedigrees with another *ABCA4* variant. In recent years, single-molecule molecular inversion probe (smMIP) based sequencing of the whole *ABCA4* gene including intronic sequences has dramatically increased positive candidate detection rates for STGD1 [32,33]. Thirty-six unresolved probands from Target 5000 presenting as STGD1 or cone-rod dystophy included in this cohort were analysed as part of a landmark study on the genetic landscape of ABCA4 in over 1000 probands, with a positive candidate detection rate of 44%. (16/36) [13]. This has increased the rate at which *ABCA4* is identified as the causal gene of STGD1 in this cohort (Figure 6). Of note from Irish participants in this study, 5 individuals were found to carry c.4539+2028C>T (p.[=,Arg1514Leufs*36]) in trans with another pathogenic *ABCA4* variant. Upon retrospective analysis, 5 further incidences of this variant were detected in previously unresolved individuals, totaling 10 participants recruited by Target 5000 carrying this variant and another pathogenic *ABCA4* variant, suggesting significant enrichment in the Irish STGD1 population.

In agreement with previous reports on Target 5000 [15,27], *ABCA4* c.5603A>T (p.Asn1868Ile) is a significant causal variant of a milder form of STGD1 in this IRD cohort. Of 110 STGD1 pedigrees with 2 positive candidate variants identified, this variant was observed 14 times. In addition, 6 STGD1 patients were found to carry this variant homozygously. *ELOVL4* and *PROM1* were determined to be the cause of STGD1 disease in 2.7% (2/112) of sequenced pedigrees. Other maculopathies that presented prominantly include best disease accounting for 9.4% (15/159) of macular dystrophies in this study, cone-rod dystrophy, and a general macular dystrophy phenotype, each accounting for almost 7% (11/159) of all maculopathy pedigrees (Figure 6). However, it is clear from Figure 6 that STGD1 is the predominant cause of macular dystrophy in Target 5000 participants.

### 3.4. Usher Syndrome

**Figure 7:**
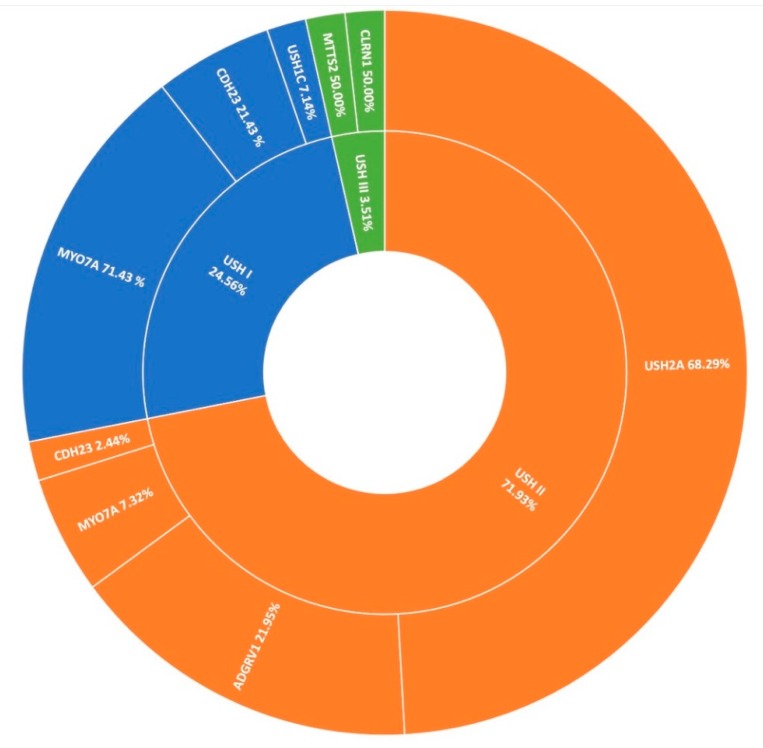
A summary of the genetic architecture of Usher Syndrome (USH) in Target 5000 participants (Table S6).

Usher syndrome is the most common manifestation of syndromic-IRD in this cohort, accounting for over 7% (78/1004) of all clinical presentations in Target 5000 (Figure 1). Usher syndrome is a form of ciliopathy characterised by RP and sensorineural hearing loss. It is generally divided into sub-groups based on severity of symptoms, ranging from most severe in Type 1 to least severe in Type 3. In total 57 pedigrees were genetically diagnosed with Usher syndrome, where 2 candidate variants were detected, with Type 2 being the most common presentation. Seventy-eight pedigrees were clinically diagnosed with Usher syndrome, yielding a positive candidate detection rate of 73% (57/78). Usher type 2 is by far the predominant sub-group in the Target 5000 cohort (41/57), most frequently caused by variants in *USH2A* (28/41) (Figure 7). Variants in *MYO7A* were the most frequent cause of Usher type 1 (10/14). Variants in *CLRN1* and *MTTS2* (1/2) contributed equally to cases of Usher Type 3 in this study, with one individual carrying a novel candidate variant in *CLRN1* (Table 2).

### 3.5. Other IRDs Encompassed by Target 5000

**Figure 8:**
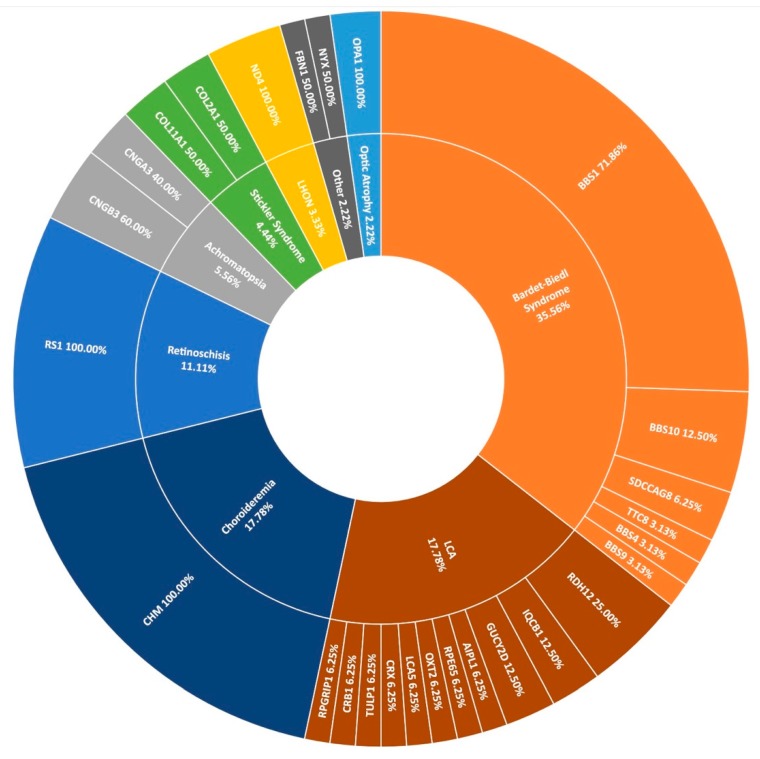
A summary of the genetic architecture of less common phenotypes encountered in the Target 5000 study. LCA: Leber Congenital Amaurosis, LHON: Leber Hereditary Optic Neuropathy (Table S7).

The second most frequent syndromic IRD clinically diagnosed in this study was Bardet-Biedl syndrome (BBS), typically characterised by polydactyly, intellectual disability, and obesity. Over 2% of pedigrees (21/1004) in the entire cohort present with BBS at the clinic (Figure 1); however, this is thought to be an under-representation, as some of those diagnosed with simplex RP are later found to have had polydactyly removed as children. These individuals are re-diagnosed with BBS following detection of two candidate variants in a BBS-associated gene and confirmation with Target 5000 affiliated ophthalmologists. In total, 14 pedigrees received a genetic diagnosis of BBS having been initially clinically diagnosed with RP. Variants in *BBS1* were the most frequent cause of this syndrome accounting for over 71% (23/32) of sequenced pedigrees. As reported previously, the most common variant is c.1169G>T (p.Met390Arg) [15]. This variant has been detected 28 times homozygously in 22 different pedigrees in this cohort. The second most frequent cause of BBS were variants in *BBS10* (4/32), one of which is believed to be a novel variant (Table 2). BBS causality is followed by *SDCCAG8* (n = 2), *BBS9*, *BBS4*, and *TTC8* (n = 1 each) in the Target 5000 cohort (Figure 8).

Many other rare forms of IRD are also included in the Target 5000 study. Sixteen pedigrees with a diagnosis with Leber congenital amaurosis (LCA) have been genotyped with a spectrum of genes implicated as positive candidates (Figure 8). Other rare IRDs examined in this study include retinoschisis (n = 10), achromatopsia (n = 5), Stickler syndrome (n = 4), Leber hereditary optic neuropathy (n = 3), and optic atrophy (n = 2), among others. In total, 90 pedigrees with positive candidate variants are encompassed by Figure 8, illustrating the wide spectrum of disease presentation included in Target 5000.

### 3.6. Novel Variants

Here we present 19 novel missense variants that, to the best of our knowledge, have not yet been associated with an IRD (Table 2). In silico tools including MetaLR, M-CAP and REVEL were utilised to predict the pathogenicity of these variants. Segregation analysis was carried out when possible and was used as evidence according to the American College of Medical Genetics and Genomics (ACMG) guidelines where appropriate [21]. Two of the variants listed here, BBS10 c.155G>A (p.Gly52Asp) and PRPH2 c.464C>T (p.Thr155Ile), had been allocated dbSNP IDs; however, this is likely due to their detection in population sequencing studies, such as gnomAD [34], where they have not previously been associated with an IRD. Ten of these variants have been classified as variants of unknown significance (VUS) although having strong in silico predicted pathogenicities. This highlights the importance of functional analysis in vitro and in vivo in order to confidently call such variants likely pathogenic.

### 3.7. RPE65

**Figure 9:**
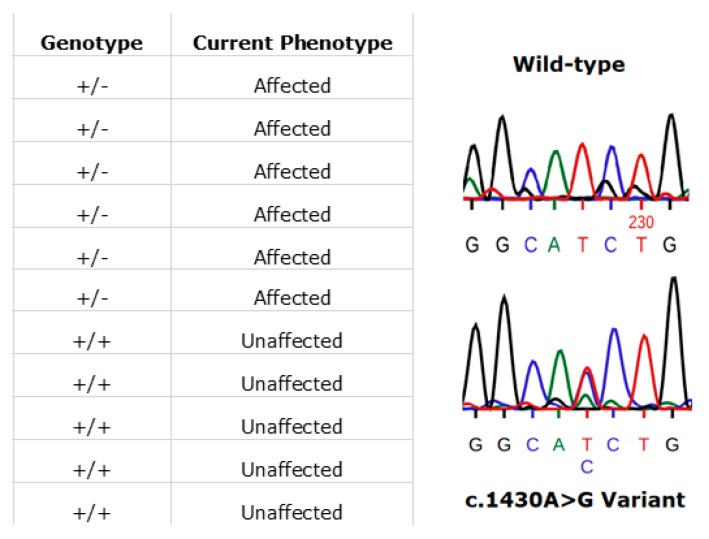
An illustration of patients harbouring the RPE65 c.1430A>G (p.D477G) variant. In this pedigree, each individual’s phenotype corresponds to the expected genotype (left image). Also shown is a representative Sanger sequence trace from an unaffected (wild-type) family member (right image, top). The variant sequence shown below is that from an affected family member (right image, bottom). Sequences were read from the sense strand so the observed change above is T>G (GGCA[T>G]CTG).

In Ireland, the prevailing RPE65 phenotype has been that associated with the specific variant, (NM_000329.2) c.1430A>G (p.Asp477Gly). The disease phenotype is comparatively much milder than *RPE65*-LCA and closely resembles the clinical manifestations of choroideremia. This amino acid position has been shown to be highly conserved across multiple species [35]. This variant is one that continues to be detected in additional cases in the ongoing Target 5000 study. Initial reports and phenotype characterisation of this mutation were based on several pedigrees originating from Ireland [35,36]. The dominant c.1430A>G (p.Asp477Gly) variant remains to be probed for, yet is undetected, in large RPE65 screening studies (n > 2000) of non-Irish ethnicity [37]. Currently in the Target 5000 cohort, there are 23 genetically confirmed affected individuals, with many patients from these pedigrees that are pending recruitment. These 23 affected patients span 7 pedigrees although likely originate from a single source as observed in previous studies [38]. An example of segregation of the c.1430A>G (p.Asp477Gly) variant in one such pedigree is shown in Figure 9.

### 3.8. FLVCR1

**Figure 10:**
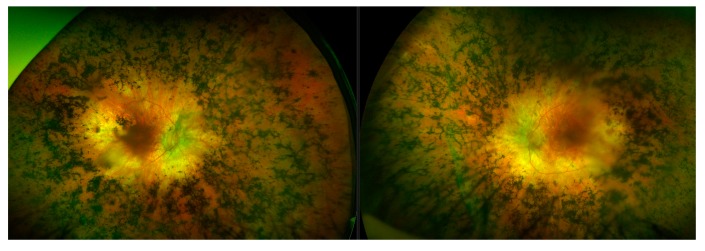
Montage of wide-field fundus images from a retinitis pigmentosa patient with a *FLVCR1* genotype. The *FLVCR1* genotype results in a phenotype (left- right eye, right- left eye) that appears to bear a resemblance to classical retinitis pigmentosa, with features such as masses of bony spicules in the periphery, attenuated blood vessels, and waxy disc pallor. There is also a relative preservation of the maculae.

Typically, variants in FLVCR1 have been associated with a neurological syndrome, posterior column ataxia with retinitis pigmentosa (PCARP; MIM: 609033) [39,40,41], and more recently a specific splice variant (c.1092 + 5G>A) has been reported multiple times to be associated with non-syndromic RP [42,43,44]. Through Target 5000, substantial evidence has been obtained that suggests the first incidence of a protein coding FLVCR1 variant c.1022A>G (p.Tyr341Cys) implicated in non-syndromic RP [45]. RP is the most common clinical diagnosis for participants in the Target 5000 study, where the clinical diagnosis of RP accounts for nearly 40% (379/1004) of total pedigrees enrolled to date (Figure 1). Patients with this variant present with typical RP, without any extraocular features (Figure 10). Since its initial detection, this variant has been observed and deemed to segregate with the condition in three additional not knowingly related families in the Target 5000 cohort. Each of the genotyped affected individuals in these three pedigrees are homozygous for this variant.

### 3.9. Choroideremia

**Figure 11:**
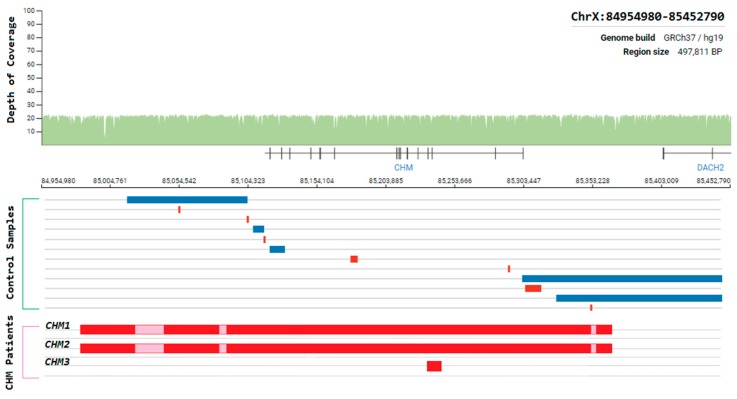
Schematic representation of the genomic region surrounding the CHM gene. Whole-genome coverage data from a control population database (https://gnomad.broadinstitute.org/). Structural variants detected from controls samples and Target 5000 patients are aligned to this region to illustrate the instability of this genomic region. Blue = duplication, Red = deletion, Pink = template positive results likely due to highly similar sequence elsewhere in the genome.

**Figure 12:**
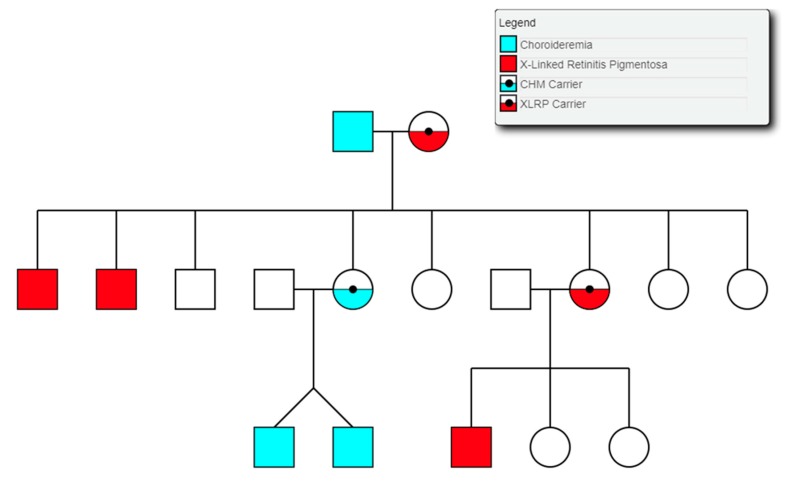
Partial pedigree tree illustrating the segregation of two X-linked retinal conditions within one family.

Choroideremia is an X-Linked recessive chorioretinal degenerative condition with progressive atrophy of various retinal cell types and the surrounding blood retinal barrier. Here we describe a novel deletion in the *CHM* gene found in two Irish pedigrees. This nearly 500 kb deletion represents the largest as yet detected IRD-associated gene deletion in Ireland (Figure 11). Two members of a large X-linked Retinitis Pigmentosa pedigree (Figure 12) clinically presented with choroideremia and tested negative for the segregating *RPGR* variant found in other affected members of this pedigree. Both males were analysed with target capture sequencing and found to possess large deletions spanning the *CHM* gene, approximating 500 kb. The observation of two IRDs in this pedigree highlights the significant value of NGS-based diagnostics for IRDs (Figure 12).

The same *CHM* deletion has also been detected in a second Irish pedigree since its initial discovery. Two additional males and two carrier females from this second pedigree were all found to be affected with progressive choroideremia. In this pedigree, the incidence of choroideremia could be traced back 5 generations, 4 of which have been assessed by the clinical team. A third Irish pedigree with a large *CHM* deletion was also detected previously as part of Target 5000 [27]. This mutation spanned approximately 6.5 kb and encompassed exons 3 and 4 of *CHM* (Figure 11). In each instance, target capture NGS detected the presence of the deletion and breakpoints were approximated by tiled PCR analyses. Interestingly, some probing PCRs in the intergenic regions surrounding *CHM* suggested that genomic DNA was present between deleted regions. Upon analysis, these PCR products were likely the result of large regions of homology that exist within this genomic area and other regions of the genome. Several structural variants in this *CHM*-proximal intergenic region have also been observed in control samples (Figure 11).

## 4. Discussion

Up to now, over 1000 individuals have been genotyped as part of Target 5000, accounting for over 20% of the estimated Irish IRD cohort. The results of this study thus far highlight the unique genetic architecture of IRDs in Ireland, cumulatively resulting in over 89 novel variants identified to date [15,27], 19 of which were missense mutations in addition to 1 novel structural variant identified in the most recent analysis (Table 2, Figure 11). With a positive candidate detection rate of almost 70% (495/710 pedigrees), Target 5000 highlights the value of sequencing the exons of 254 IRD-associated genes as well as some known pathogenic intronic regions. This is consistent with other sequencing studies of various IRD cohorts, which report a diagnostic yield of between 38–75% [17,46,47,48,49]. This range can be expected due a number of factors. Firstly, deep-phenotyping to accurately categorise IRDs greatly assists with genotype-phenotype assessment. Assessor variability will exist between clinics and studies, contributing greatly to the variability of diagnostic yield. Secondly, there are differences between the numbers of genes and regions sequenced in these cohorts, where smaller gene panels will not capture some of the known genes associated with IRDs [46]. Lastly, the employment of additional analyses such as WES or WGS in other studies has increased the detection of candidate variants in previously unresolved cases [18,49,50,51]. The candidate variant detection rate of Target 5000 demonstrates that target capture sequencing is a cost and time-effective first-tier approach for genetic screening of those affected by IRDs in Ireland.

This study also highlights the importance of an accurate genetic diagnosis in addition to a clinical diagnosis. Genetic diagnoses continue to become more relevant as gene-based medicines move towards the forefront of IRD treatment given a first approved gene therapy for an IRD and an array of gene therapies for other forms of IRD in clinical and preclinical development. Furthermore, genetic diagnoses facilitate a better understanding of disease progression and manifestation for both patients and clinicians.

Stargardt disease (STGD1) is an autosomal recessive disorder caused almost exclusively by variants in the ATP-binding cassette subfamily A member 4 (*ABCA4*) gene (MIM: 601691) [52]. It is the most frequent form of macular dystrophy with an estimated prevalence of approximately 1 in 10,000 individuals [53]. STGD1 is characterised by progressive bilateral central vision loss, colour vision defects, delayed dark adaptation, and flecks in the retinal pigmentary epithelium [54]. A spectrum of disease severity underlies STGD1, with age of onset and progression governed by the specific combination of variants an individual carries, highlighting again the value of a specific genetic diagnosis for disease progression and risk assesment. An extensive list of 5962 likely pathogenic *ABCA4* variants in 3928 cases was published in 2017 [55], providing a valuable resource to aid in the interpretation of variants discovered as part of this study. In addition to coding variants captured, it has become increasingly clear that many of the unresolved one allele only (Figure 2) cases may likely carry a pathogenic intronic variant where the disease is typically associated with a recessive inheritance pattern. This has been elegantly demonstrated by deep intronic variants in *ABCA4* causing STGD1, such as c.4539+2028C>T (p.[=,Arg1514Leufs*36]). This variant has been observed as a candidate in 10 Target 5000 individuals, of which 5 were disovered as part of a landmark study of *ABCA4*-linked STGD1 employing smMIPs based whole gene sequencing [13] and 5 of which were discovered through target capture sequencing runs. This highlights a significant enrichment of this variant in the Irish IRD patient cohort. c.4539+2028C>T (p.[=,Arg1514Leufs*36]) was previously functionally analysed in photoreceptor precursor cells, and was shown to result in a 345 nucleotide pseudoexon inclusion due to strengthening of exonic splice enhancers. It is notable that such variants are an attractive target for antisense oligonucleotide-based splice correction therapy [56].

Identified variants proximal to the *RHO* p.Met207 region appear predominant in our sub-cohort of autosomal dominant RP patients, as reported in Section 3.2. Amino acids in this region of the protein form part of the fifth transmembrane domain of rhodopsin. This domain has been shown to be vital to retinal binding in bovine rhodopsin [57]. It is possible that undesirable changes in the protein folding of this region result in steric hindrance that prevents the apoprotein from successfully binding retinal. This impaired binding capacity may possibly underpin the reason for the prevalence of pathogenic variants found in the coding region of this protein domain.

Feline leukaemia virus subgroup C cellular receptor 1 (*FLVCR1*; MIM: 609144) is a transmembrane protein involved in erythropoiesis and heme transport [58]. *FLVCR1* was first discovered for its role in aplastic anaemia in domestic cats and subsequently erythroblast destruction in vitro [59]. This was also the first study that theorised that *FLVCR1* was a receptor for an organic anion and identified its component domains as strikingly similar to other ancient Major Facilitator Superfamily (MFS) members. In a later study it was shown this receptor protein provided the first description of a mammalian heme transporter [60]. The same group proceeded to investigate its effect in knockout mouse models, which resulted in embryonic lethality [58]. Candidate variant detection in *FLVCR1* has significantly increased in recently recruited pedigrees. It is notable the c.1022A>G (p.Tyr341Cys) variant is only the second disease-associated variant to be found in *FLVCR1* associated with RP without posterior column degeneration [43]. Moreover, it is the first protein coding variant found in this gene to be affiliated with non-syndromic RP [45]. There were no extraocular features associated with this variant (Figure 10). It is possible that the distinct families in this cohort with this variant share a common ancestry. However, they do not share any rare variants in the regions captured and originate from distinct geographical locations in Ireland. The age of onset for the symptoms of ataxia in PCARP has been reported as typically in the third decade of life. Of note in the Target 5000 cohort, all patients carrying this variant have reached this age, with the oldest patient currently in their seventh decade of life. It is unclear yet as to whether the disease pathology associated with this variant will remain completely non-syndromic throughout the entirety of a patient’s lifespan or will result in a milder, later onset of additional symptoms compared to other pathogenic variants found in this gene. Fortunately, the small size of this gene (2.6 kb) in principle makes it suitable for inclusion in AAV vectors for use as a gene therapy. Such a therapy may help to alleviate the toxic effects of intracellular free-heme [39]. The serotype of AAV could be chosen based on the presence or absence of systemic phenotypes. For example, if only the retina was to be targeted, an AAV 2/5 or 2/8 might be effective serotypes; on the other hand, if the whole central nervous system was to be targeted, then AAV9 or AAVB1 would be more valuable [61]. Many additional bespoke serotypes are now emerging as a result of site-directed evolution of AAV serotypes [62,63,64,65]. Given the recent results here on the role of FLVCR1 in this form of non-syndromic RP, FLVCR1 along with other IRD genes, becomes an increasingly interesting candidate for exploration of AAV-mediated gene therapies.

The RPE65 enzyme is active in the retinoid cycle that recycles retinoids in the RPE, which are utilised by photoreceptor cells. Mutations in the RPE65 gene were initially found to be causative of some cases of autosomal recessive LCA, a severe and clinically distinct IRD [66,67,68]. This was subsequently expanded to include a milder, dominant form of retinopathy and more recently, to include less severe autosomal recessive retinopathies [35,37]. Luxturna, the first ocular gene therapy approved by the FDA, is for biallelic RPE65 retinal disease [16]. RPE65 c.1430A>G (p.Asp477Gly) remains the predominant RPE65 genotype observed in the Irish population. This variant has since been detected in other population studies alongside the clinical presentation of similar phenotypes, largely described as a choroideremia phenocopy [36,38]. However, until recently the exact disease mechanism caused by this variant has evaded researchers. It was initially believed that this variant produced an abnormal protein structure that may affect enzymatic function [35]. More recently, it has been shown in a mouse model that although this variant is not located in a critical functional domain, the variant induces sufficient change to alter the physiochemical properties of the p.D477 loop to produce an aggregation-prone surface. This surface gains the aberrant function of enabling abnormal protein-protein interactions [69]. One hypothesised type of interaction is with ubiquitin ligases, which would likely result in proteasomal degradation as seen in several other RPE65 mutations [70]. This theory may support the variability observed phenotypically as severity might then become dependent on several other factors such as ubiquitination rates and proteasome response. It is of note that aberrant RNA splicing has been shown to have a major pathogenic effect in a knock-in mouse model with this variant [71]. Given the success of the gene therapy treatment of biallelic RPE65 retinopathies with Luxturna [16], an AAV-based therapy, it is interesting to speculate whether the therapy might have any utility for monoallelic dominant RPE65 cases.

The *CHM* gene hosts the largest collection of pathogenic structural variants detected in any IRD-related gene as part of the Target 5000 study to date. The presence of several structural variants in the general population may also indicate a natural instability in this genomic region. Additionally, female carriers of *CHM* mutations typically show mild stationary signs with no symptoms, while males are severely affected. In this instance, some females were more severely affected than expected with advanced signs of degeneration and progressive visual decline. One female carrier showed radial pigmentation at the retinal pigment epithelium level and scattered choroidal atrophy, whereas another showed more marked reactive pigmentation and extensive symmetrical atrophy of the choroid and outer retina. These manifestations were apparent both anatomically and functionally. A more severe phenotype was also associated with whole *CHM* gene deletion in a previous genotype-phenotype choroideremia study [72]. However, in this study, the phenotype was described as choroideremia plus, due to additional syndromic features. These additional symptoms, such as deafness, were attributed to the extent of the deletion encompassing several surrounding genes, which is not the case for the whole gene deletion patients described here.

Going forward with the Target 5000 study, more efforts will be focused on the detection of additional variants in unresolved IRD cases and also in the functional assessment of variants of unknown significance identified thus far. In the age of budding emerging gene therapies, it has never been more important to stringently identify the pathogenic variants that underpin a condition. It is therefore vital that the variants located outside of exonic regions causing disease pathologies are detected, and that these participants are also considered for treatment, should an applicable gene therapy become available in the future. A whole exome or whole genome approach will be more extensively employed in the future to increase the identification of novel variants. Recent studies have highlighted that molecular diagnostic yield is increased when WGS is employed by comparison with other methods [51]. It is expected that for many of the currently unresolved cases, pathogenic variants may be present in deep-intronic, promoter, enhancer regions or in genes that are not yet associated with an IRD [73,74]. Recent studies such as that in STGD1, highlighting the relative prevalence of deep intronic mutations in the *ABCA4* gene, attest to the need for more extensive sequence analysis [13]. These regions have been very poorly covered in patients screened by target capture protocols, unless specifically designed to do so. Additionally, WGS data will offer higher resolution in terms of data associated with copy number abnormalities and large structural rearrangements. It has been reported that there are over 1300 CNVs associated with IRD genes reported in the literature [75]. Exon-based target capture methods have a significantly reduced capacity to detect CNVs and SVs. This is due in part to the range of read-depth at captured regions and the inability to detect breakpoints that occur outside of captured loci. Notably, similarly targeted methods, such as non-WES/WGS studies, which have specifically probed intronic and exonic regions of genes have been shown to successfully detect large structural variants [13]. This illustrates that the enormous data volumes generated by WGS studies may be surplus to requirement if the SVs/CNVs to be searched for are relevant to a small genomic region.

Table 2 illustrates that many variants with seemingly strong evidence of pathogenicity may still be classified as VUS. Variants that are currently of unknown significance likely to alter enzyme function, such as those in *RPE65* [76], may be applicable to assessment in vitro by employing enzyme function assays. Alternatively, if predicted to perturb splicing having undergone in silico interrogation [77], these variants could be assessed by midigene analysis. Midigene analysis refers to the incorporation of variants of interest into vectors containing several exons of the relevant IRD gene, enabling the in vitro interrogation of the effect(s) of that variant directly at an RNA level. This approach is particularly effective for testing exonic and intronic variants that are likely to disrupt correct splicing, as these variants will have a distinct impact on the RNA product when compared to the wild-type equivalent. This method is also particularly useful for the interpretation of variants that are located within introns, yet outside of canonical splice sites, and additionally to examine rare synonymous exonic variants proximal to exon junctions [32,78].

A viable in vivo strategy for the assessment of novel missense variants is to introduce these VUS into model organisms with similar or conserved genes. The significant advantages of such model organisms is the ability to rapidly generate models of disease [79]. This is particularly relevant currently, as recent advances in genome editing technologies has made this approach more readily accessible and successful than ever before [80]. Although not all model organisms fully represent the visual system that exists in humans, many organisms can offer useful information ranging from protein mislocalisation [81] to optokinetic response [82]. By introducing a VUS into the appropriate system, additional evidence can be gained to better evaluate the functional impact of the VUS in human ocular pathologies.

Thus far, genetic analysis of IRD patients has helped to resolve ambiguous phenotypes and to identify causative mutations in nearly 70% (495/710) of sequenced pedigrees. The continuous expansion of our cohort has enabled us to better interrogate the sequencing data and interpret the potential pathogenicity of novel variants when detected. In addition to this, the growing body of data from NGS studies of IRDs globally should facilitate better correlations between genotype and phenotype and further refine methods for diagnoses and prognoses. Furthermore, whole gene and WGS analyses are highlighting the significant role of non-coding variants as causative of some IRDs. Target 5000 aims to provide actionable outcomes empowering patients with genetic diagnoses and potentially future access to clinical trials or approved treatments where appropriate. Given the rapid development of the field of ocular therapeutics, it is clear that genetically characterising those affected by an IRD has become a diagnostic imperative of the utmost importance.

## Figures and Tables

**Table 1:** Table of typical assessments, instruments, and manufacturers routinely used as part of Target 5000 recruitment.

Analyses	Method	Manufacturer
Best-corrected visual acuity	Revised 2000 early treatment diabetic retinopathy study (ETDRS) charts	Precision Vision, La Salle, IL, USA
Colour vision	Lanthony desaturated D-15 panel under standardised lighting conditions	Gulden Ophthalmics, Elkins Park, PA, USA
Peripheral visual fields	Goldmann perimeter 940 (iv4e, i4e and 04e targets)	Haag-Streit AG, Köniz, Switzerland
Full field electroretinograms	Roland Consult RETI-port retiscan.	Brandenburg an der Havel, Germany
Fundus colour and autofluorescence photography	Topcon CRC50DX/Optos Daytona	Topcon Great Britain Ltd., Berkshire, England/Optos plc, Dunfermline, Scotland
Spectral domain optical coherence tomography	Cirrus HD-OCT	Carl Zeiss Meditec, Berlin, Germany

**Table 2:** List of 19 novel missense variants discovered in Target 5000. This table does not include novel structural variants reported below. To the best of our knowledge, mutations listed here have not been previously associated with an IRD. American College of Medical Genetics and Genomics (ACMG) guidelines have been applied to all variants [21], with modifications made in accordance with recommendations by Romanet et al. [23]. (S): reclassified as strong evidence. VUS: Variant of unknown significance.

Gene	Condition	Transcript ID	Chr	Genomic Location	Nucleotide Change	Protein Change	ACMG Criteria	ACMG Classification	MetaLR Score	M-CAP Score	REVEL Score	Observed With
ABCA4	Stargardt Disease	NM_000350.2	chr1	g.94062649	c.1865G>A	p.Ser622Asn	PM2, PP3, PP4(S)	Likely Pathogenic	0.668	0.065	0.279	c.161G>A
ABCA4	Macular Dystophy (Recessive)	NM_000350.2	chr1	g.94111517	c.223T>C	p.Cys75Arg	PM2, PM5, PP3	VUS	0.995	0.711	0.967	c.4253+43G>A
ABCA4	Stargardt Disease	NM_000350.2	chr1	g.94029516	c.4468T>C	p.Cys1490Arg	PM2, PP3, PP4(S)	Likely Pathogenic	0.952	0.723	0.932	c.3305A>T
ABCA4	Macular Dystophy (Recessive)	NM_000350.2	chr1	g.94014674	c.5329A>G	p.Met1777Val	PM2, PP3	VUS	0.663	0.066	0.758	c.1037A>C
ABCA4	Stargardt Disease	NM_000350.2	chr1	g.93996182	c.6743T>C	p.Phe2248Ser	PM2, PP3, PP4(S)	Likely Pathogenic	0.861	0.528	0.935	c.302+68C>T
AHI1	Retinitis Pigmentosa (Recessive)	NM_001134830.1	chr6	g.135428670	c.2582G>A	p.Gly861Glu	PM2, PM3, PP1, PP3	Likely Pathogenic	0.583	0.159	0.816	c.1267C>T
AIPL1	Leber Congenital Amaurosis	NM_014336.4	chr17	g.6425759	c.856G>C	p.Asp286His	PM2, PM3, PP1, PP3	Likely Pathogenic	0.877	0.290	0.791	c.834G>A
BBS10	Retinitis Pigmentosa (Recessive)	NM_024685.3	chr12	g.76348204	c.155G>A	p.Gly52Asp	PM1, PM2, PP3	VUS	0.527	0.050	0.591	c.2119_2120delGT
BEST1	Best Vitelliform Macular Dystrophy (Dominant)	NM_001139443.1	chr11	g.61955783	c.133C>G	p.Arg45Gly	PM2, PP3, PP4(S)	Likely Pathogenic	0.966	0.699	0.945	/
BEST1	Best Vitelliform Macular Dystrophy (Dominant)	NM_001139443.1	chr11	g.61955157	c.23A>G	p.Tyr8Cys	PM2, PP3, PP4(S)	Likely Pathogenic	0.976	0.710	0.917	/
CLRN1	USH Type III	NM_001195794.1	chr3	g.150928039	c.635A>G	p.His212Arg	PM2, PM3, PP1, PP3	Likely Pathogenic	0.573	0.055	0.799	c.118T>G
CNGA3	Achromatopsia	NM_001298.2	chr2	g.98391904	c.607T>C	p.Trp203Arg	PM2, PP3	VUS	0.960	0.316	0.950	c.1694C>T
MYO7A	USH Type I	NM_000260.3	chr11	g.77192227	c.4101C>G	p.Ile1367Met	PM2, PP3	VUS	0.542	0.220	0.672	c.2904G>T
PEX7	Uncategorised Syndrome	NM_000288.3	chr6	g.136822742	c.77C>T	p.Pro26Leu	PM2, PP3, PP4	VUS	0.962	0.947	0.864	c.875T>A
PRPF31	Retinitis Pigmentosa (Simplex)	NM_015629.3	chr19	g.54124558	c.757G>A	p.Gly253Arg	PM2, PP3	VUS	0.946	0.911	0.957	/
PRPH2	Retinitis Pigmentosa (Dominant)	NM_000322.4	chr6	g.42721878	c.457A>G	p.Lys153Glu	PM2, PP3	VUS	0.628	0.180	0.864	/
PRPH2	Retinitis Pigmentosa (Dominant)	NM_000322.4	chr6	g.42721871	c.464C>T	p.Thr155Ile	PM2, PP3	VUS	0.518	0.100	0.649	/
RDH5	Fundus Albipunctatus	NM_001199771.1	chr12	g.55721276	c.92T>C	p.Val31Ala	PM2, PP3	VUS	0.670	0.143	0.737	c.712G>T
USH2A	USH Type II	NM_206933.2	chr1	g.216247097	c.2297G>A	p.Cys766Tyr	PM2, PM3, PM5, PP3	Likely Pathogenic	0.945	0.669	0.952	c.2299delG

## Supplementary Materials

The following are available online at https://www.mdpi.com/2073-4425/11/1/105/s1, Table S1: Primers used to probe exonic regions of CHM (MIM: 300390; ChrX: 85861180-86047558) and the neighbouring genes, POF1B (MIM: 300603; ChrX: 85277396-85379709) and DACH2 (MIM: 300608; ChrX: 86148451-86832602) as well as the intergenic space between. Table S2: Tabular summary of the clinical presentation of 1004 recruited Target 5000 pedigrees. RP: Retinitis Pigmentosa, FFM: Fundus Flavimaculatus, MD: Macular Dystrophy, CRD: Cone-Rod Dystrophy, RCD: Rod-Cone Dystrophy, LCA: Leber Congenital Amaurosis, EOSRD: Early-Onset Retinal Degeneration, BBS: Bardet-Biedl Syndrome, CSNB: Congenital Stationary Night Blindness, Table S3: Tabular summary of the diagnostic yield rates for 710 Target 5000 pedigrees utilising target capture next-generation sequencing of the exonic regions of over 250 genes and previously identified pathogenic intronic variants, Table S4: Tabular summar of the genetic architecture of retinitis pigmentosa (RP) in Target 5000 participants, Table S5: Tabular summary of the genetic architecture of macular dystrophies in Target 5000 participants. CRD: Cone-Rod Dystrophy FA: Fundus Albipunctatus, Table S6: Tabular summary of the genetic architecture of Usher Syndrome (USH) in Target 5000 participants, Table S7: Tabular summary of the genetic architecture of less common phenotypes encountered in the Target 5000 study. LCA: Leber Congenital Amaurosis, LHON: Leber Hereditary Optic Neuropathy, Figure S1: The available age distribution of recruited Target 5000 participants, Figure S2: Sequencing coverage from three affected X-linked retinitis pigmentosa individuals (H41, P1 and K1) from three distinct pedigrees as seen on IGV software. Alligned to this region is sequencing primers (R4,R7,R8,R9,F10 and R5) and PCR primers (F3,R6 and P9) as shown by forward (blue) and reverse (red) orientations.
