# Supplementary material for: Findings from a Genotyping Study of over 1000 People with Inherited Retinal Disorders in Ireland
Source: Genes (Basel). 2020 Jan 16;11(1):105. doi: 10.3390/genes11010105 (PMC7016747; doi:10.3390/genes11010105)
Supplement: Supplementary file 1 [file genes-11-00105-s001.zip › Supplementals/Legends for Supplementary Materials.docx]

**Supplementary Materials**

**Table S1.** Primers used to probe exonic regions of CHM (MIM: 300390; ChrX: 85861180-86047558) and the neighbouring genes, POF1B (MIM: 300603; ChrX: 85277396-85379709) and DACH2 (MIM: 300608; ChrX: 86148451-86832602) as well as the intergenic space between.

**Table S*2*.** Tabular summary of the clinical presentation of 1004 recruited Target 5000 pedigrees. RP: Retinitis Pigmentosa, FFM: Fundus Flavimaculatus, MD: Macular Dystrophy, CRD: Cone-Rod Dystrophy, RCD: Rod-Cone Dystrophy, LCA: Leber Congenital Amaurosis, EOSRD: Early-Onset Retinal Degeneration, BBS: Bardet-Biedl Syndrome, CSNB: Congenital Stationary Night Blindness

**Table S3.** Tabular summary of the diagnostic yield rates for 710 Target 5000 pedigrees utilising target capture next-generation sequencing of the exonic regions of over 250 genes and previously identified pathogenic intronic variants.

**Table S4.** Tabular summar of the genetic architecture of retinitis pigmentosa (RP) in Target 5000 participants.

**Table S*5*.** Tabular summary of the genetic architecture of macular dystrophies in Target 5000 participants. CRD: Cone-Rod Dystrophy FA: Fundus Albipunctatus.

**Table S6.** Tabular summary of the genetic architecture of Usher Syndrome (USH) in Target 5000 participants.

**Table S7.** Tabular summary of the genetic architecture of less common phenotypes encountered in the Target 5000 study. LCA: Leber Congenital Amaurosis, LHON: Leber Hereditary Optic Neuropathy.

**Figure S1.** The available age distribution of recruited Target 5000 participants.

**Figure S2.** Sequencing coverage from three affected X-linked retinitis pigmentosa individuals (H41, P1 and K1) from three distinct pedigrees as seen on IGV software. Alligned to this region is sequencing primers (R4,R7,R8,R9,F10 and R5) and PCR primers (F3,R6 and P9) as shown by forward (blue) and reverse (red) orientations.
