# Supplementary figures and images for: Findings from a Genotyping Study of over 1000 People with Inherited Retinal Disorders in Ireland
Source: Genes (Basel). 2020 Jan 16;11(1):105. doi: 10.3390/genes11010105 (PMC7016747; doi:10.3390/genes11010105)

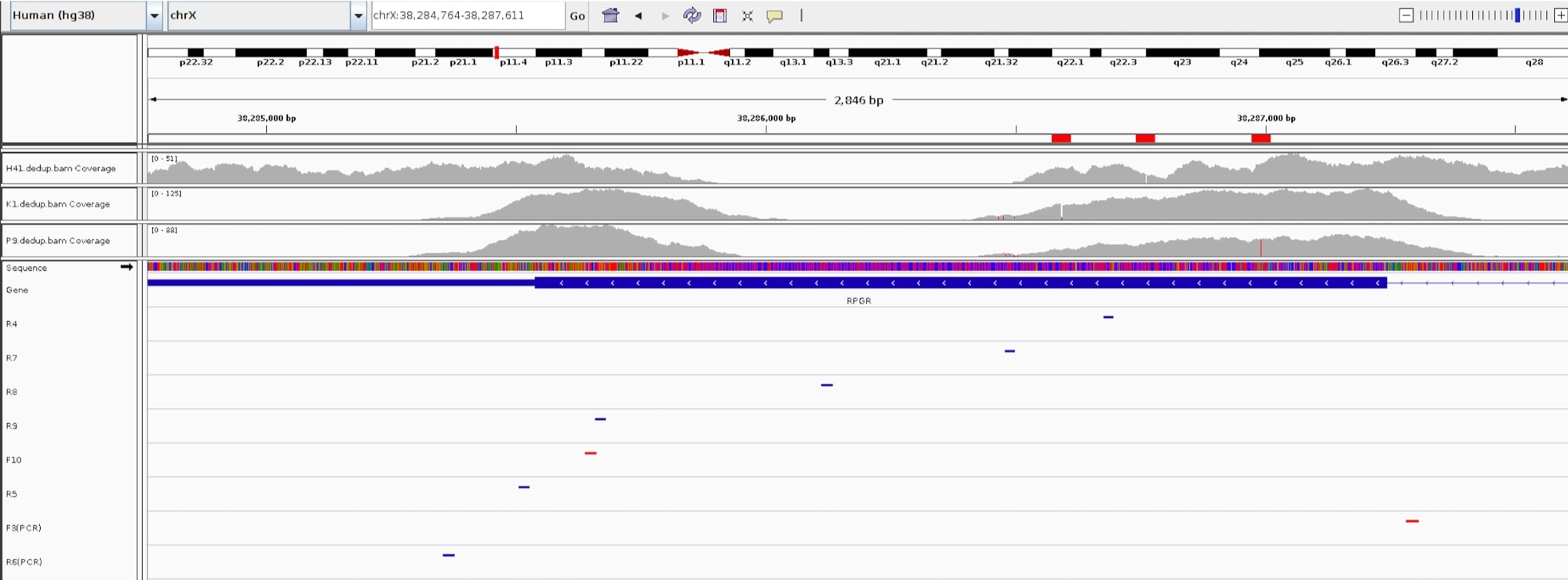

Supplement: Supplementary file 1 [file genes-11-00105-s001.zip › Supplementals/Figure S2.jpg]

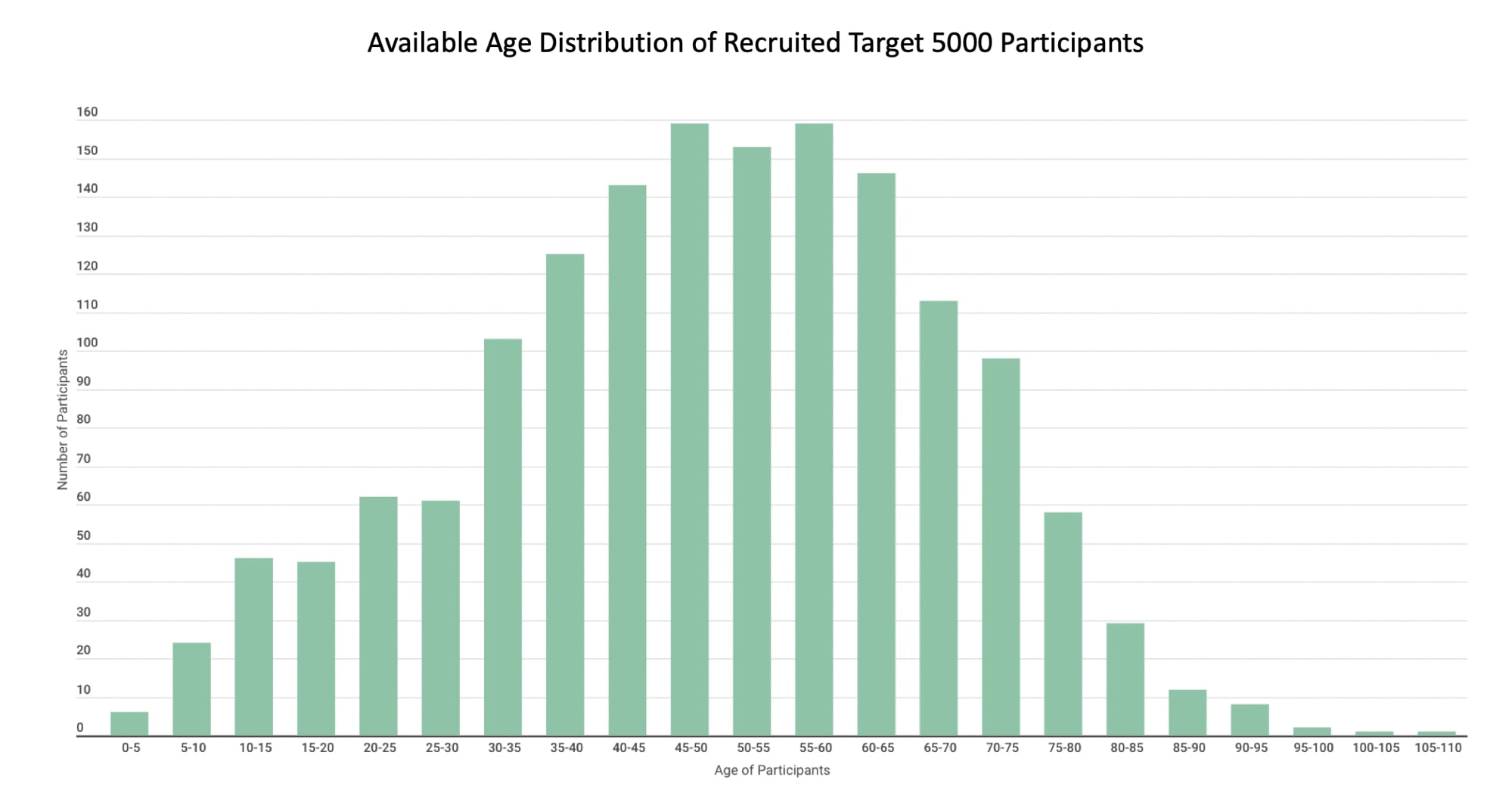

Supplement: Supplementary file 1 [file genes-11-00105-s001.zip › Supplementals/Figure S1.jpg]
